# Changes in depression and anxiety through mindfulness group therapy in Japan: the role of mindfulness and self-compassion as possible mediators

**DOI:** 10.1186/s13030-019-0145-4

**Published:** 2019-02-18

**Authors:** Toru Takahashi, Fukiko Sugiyama, Tomoki Kikai, Issaku Kawashima, Siqing Guan, Mana Oguchi, Taro Uchida, Hiroaki Kumano

**Affiliations:** 1 Graduate School of Human Sciences, Waseda University, 2-579-15, Mikajima, Tokorozawa-shi, Saitama 359-1192 Japan; 2 Japan Society for The Promotion of Science, 5-3-1, Kojimachi, Chiyoda-ku, Tokyo 102-0083 Japan; 3 Faculty of Human Studies, Bunkyo Gakuin University, 1196, Kamekubo, Fujimino-shi, Saitama 356-8533 Japan; 4 ATR Brain Information Communication Research Laboratory Group, 2-2-2, Hikaridai, Seika-cho, Soraku-gun, Kyoto 619-0288 Japan; 5 School of Human Sciences, Waseda University, 2-579-15, Mikajima, Tokorozawa-shi, Saitama 359-1192 Japan; 6 Faculty of Human Sciences, Waseda University, 2-579-15, Mikajima, Tokorozawa-shi, Saitama 359-1192 Japan

**Keywords:** Mindfulness, Depression, Anxiety, Mind wandering, Self-compassion, BIS/BAS

## Abstract

**Background:**

Mindfulness-based interventions are increasingly being implemented worldwide for problems with depression and anxiety, and they have shown evidence of efficacy. However, few studies have examined the effects of a mindfulness-based group therapy based on standard programs for depression and anxiety until follow-up in Japan. This study addresses that gap. Furthermore, this study explored the mechanisms of action, focusing on mindfulness, mind wandering, self-compassion, and the behavioral inhibition and behavioral activation systems (BIS/BAS) as possible mediators.

**Methods:**

We examined 16 people who suffered from depression and/or anxiety in an 8-week mindfulness group therapy. Measurements were conducted using questionnaires on depression and trait-anxiety (outcome variables), mindfulness, mind wandering, self-compassion, and the BIS/BAS (process variables) at pre- and post-intervention and 2-month follow-up. Changes in the outcome and process variables were tested, and the correlations among the changes in those variables were explored.

**Results:**

Depression and anxiety decreased significantly, with moderate to large effect sizes, from pre- to post-intervention and follow-up. In process variables, the observing and nonreactivity facets of mindfulness significantly increased from pre- to post-intervention and follow-up. The nonjudging facet of mindfulness and self-compassion significantly increased from pre-intervention to follow-up. Other facets of mindfulness, mind wandering, and the BIS/BAS did not significantly change. Improvements in some facets of mindfulness and self-compassion and reductions in BIS were significantly correlated with decreases in depression and anxiety.

**Conclusions:**

An 8-week mindfulness group therapy program may be effective for people suffering from depression and anxiety in Japan. Mindfulness and self-compassion may be important mediators of the effects of the mindfulness group therapy. Future studies should confirm these findings by using a control group.

**Trial registration:**

University Hospital Medical Information Network (UMIN CTR) UMIN000022966. Registered July 1, 2016, https://upload.umin.ac.jp/cgi-open-bin/ctr/ctr_view.cgi?recptno=R000026425

## Background

Mindfulness-based intervention has gained increased attention in mental health research. Jon Kabat-Zinn [1], who developed mindfulness-based stress reduction (MBSR), defined mindfulness as “the awareness that emerges through paying attention on purpose, in the present moment, and nonjudgmentally to the unfolding of experience moment by moment” [2]. Two standard mindfulness approaches exist: MBSR and mindfulness-based cognitive therapy (MBCT) [3]. Both have been developed as 8-week programs where practitioners observe their sensations, emotions, and thoughts with a non-reactive attitude. These programs have been shown by many studies to be effective for a wide variety of psychological problems, including depression and anxiety [4].

However, few studies have been used to assess the effects of mindfulness group therapy based on a standard 8-week program for depression and/or anxiety in Japan. Yanagisawa, Fujita, Mizuno, Adachi, Yoshino, and Takazawa [5] conducted a rare practical study that monitored a group program based on MBCT for Japanese psychiatric hospital outpatients who had been diagnosed with depression, anxiety disorders, fibromyalgia, and schizophrenia. Yanagisawa et al. [5] found a significant decrease in depression and a significant increase of quality of life in their single-arm study. However, they did not monitor changes in anxiety or show the results of follow-up (FU) assessment. Therefore, in this study we aimed to evaluate the possible effects of an 8-week mindfulness group therapy program for depression and anxiety until a 2-month FU. It will necessary to further clarify the changes in depression and anxiety through the mindfulness program in future randomized controlled trials.

The mechanisms of action for 8-week mindfulness programs have been studied, and many candidates for mediator variables have been proposed. Recent systematic reviews [6, 7] have presented multiple studies showing that increased mindfulness and reduced negative repetitive thinking mediate improved clinical outcomes. However, those mediation models did not show complete mediation; that is, those mediators explained only a part of variance in clinical outcomes. Therefore, we aimed to explore other possible mediators that have thus far lacked sufficient investigation. We focused on three cognitive, emotional, and behavioral tendencies as process variables: mind wandering, self-compassion, and behavioral inhibition and behavioral activation systems (BIS/BAS).

First, we considered that mindfulness group therapy programs may decrease mind wandering, which in turn leads to decreased depression and anxiety. Mind wandering has been described as “drifting away from a task toward unrelated inner thoughts, fantasies, feelings, and other musings” [8]. Several studies have shown that mind wandering is related to depression [9], reduced psychological health [10], and reduced levels of happiness [11]. In a study by Mrazek, Smallwood, and Schooler [12], self-reported mindfulness was negatively correlated with mind wandering, and mindfulness meditation reduced mind wandering in an experimental setting. In addition, mind wandering was found to mediate the relationship between self-reported mindfulness and negative moods in a cross-sectional study [13]. Thus, mind wandering is thought to be a mediating variable for improvement in depression and anxiety through mindfulness group therapy. However, this has not yet been examined.

Additionally, it was considered that mindfulness group therapy programs may increase self-compassion, in turn leading to decreased depression and anxiety. Neff [14] described self-compassion as “being touched by and open to one’s own suffering, not avoiding or disconnecting from it, generating the desire to alleviate one’s suffering and to heal oneself with kindness.” It was found that self-compassion is related to reduced depression, trait anxiety, and greater life satisfaction [14]. It has also been shown that the effects of MBCT on recurrent depression are mediated by enhancements in self-compassion and mindfulness [15]. However, few studies have shown an effect of mediation through self-compassion for the reduction of depression and anxiety in 8-week mindfulness group therapy, according to recent systematic reviews [6, 7].

In addition, we considered that mindfulness-based group therapy programs may decrease BIS and increase BAS, leading to decreased depression and anxiety. The BIS is an aversive motivational system that is sensitive to signals of punishment, nonreward, and novelty. It inhibits behaviors that may lead to negative or painful outcomes, and this inhibits movement toward goals [16]. The BIS is related to trait anxiety [17] and depression [18]. The BAS is an appetitive motivational system that is sensitive to signals of reward, nonpunishment, and escape from punishment [16]. The BAS is related to positive affectivity [16] and lowered levels of depression [18]. Some factors of mindfulness are related to lower BIS and higher BAS [19]. Thus, decreases in the BIS and increases in the BAS are thought to mediate the changes in depression and anxiety brought about by mindfulness group therapy programs, but this has not yet been examined.

The present study tested the following hypotheses: As a result of taking part in a mindfulness program, 1) depression and anxiety will decrease (outcome variables), and such decreases will last for at least 2 months FU; 2) mindfulness, self-compassion, and the BAS will show increases, while mind wandering and the BIS will show decreases (process variables), and these changes last for at least 2 months FU; and 3) changes in outcome and process variables will be correlated, and we will explore the correlations between them.

## Methods

### Participants

Participants who complained of depression and/or anxiety were recruited using the website of the clinic where the program was to take place, social networking services, and leaflets distributed at the clinic. The criteria for exclusion comprise: 1) being diagnosed with or suspected of personality disorder, eating disorder, bipolar 1 disorder, bipolar 2 disorder (manic phase), schizophrenia, autism spectrum disorder, attention-deficit/hyperactivity disorder, obsessive compulsive disorder, or PTSD, and 2) being evaluated as likely to face difficulties in participating in group therapy (e.g., presenting high suicidal tendencies, problems with communication, etc.). Before participating in the group therapy program, subjects were interviewed on their symptoms, problems, the history of these, and their reasons for participating in the program, and a Mini-International Neuropsychiatric Interview (M.I.N.I.) [20] was administered. Questions on alcohol or substance dependence and abuse and antisocial personality disorder were omitted, because the purpose of the M.I.N.I. was to assess depression, anxiety disorders, and the disorders included in the exclusion criteria in this study. Then, the mindfulness group therapy and the study were explained. The interview described took from 60 to 90 min. After these steps were completed, the participants provided written informed consent.

An a priori power calculation showed that a sample of at least 12 participants was required to detect large effect sizes (*η*^2^ = 0.14), using a repeated one-way ANOVA (alpha = 0.05, power = 0.8, correlations among repeated measures = 0.5). This analysis was based on large effect sizes for depression and anxiety and correlations among those repeated measures in our preliminary intervention. The contents and participants of the intervention were similar to those of the current study. In all, 24 people applied to take part in the 8-week mindfulness program. They all received an individual interview, and 17 participated in the study. However, responses to self-reported scales at post-intervention and FU were not available from one participant, and therefore, we were only able to analyze data from 16 people (4 men, 12 women; age, 41.3 ± 9.2).

The results of M.I.N.I. are shown in Table 1. Although one participant reported having obsessive-compulsive disorder in the M.I.N.I., that person was permitted to participate in the program because, based on the individual interview, the DSM criteria for obsessive-compulsive disorder were not met. Each participant paid 10,000 Japanese yen to receive the therapy.

**Table 1:** The results of M.I.N.I

	Number of cases
MD with melancholic features & lifetime PD & AG	1
MD with melancholic features & past MD & AG & GAD & Moderate suicidal tendency	1
Dysthymia & AG & Mild suicidal tendency	1
PD	1
AG	1
Lifetime PD & AG	2
AG & OCD	1
AG & Moderate suicidal tendency	1
Moderate suicidal tendency	1
Not categorized by any diagnosis^a^	6

Note. *MD* major depression, *PD* panic disorder, *AG* agoraphobia, *OCD* obsessive compulsive disorder, *GAD* generalized anxiety disorder

^a^Not evaluated as having any diagnosis or problem by M.I.N.I

### Intervention

The group therapy was conducted at a psychiatry and psychosomatic-medicine clinic in Saitama Prefecture, Japan. This study was conducted from June 2017 to February 2018. The program conducted was based on MBSR [1] and MBCT [3]. Table 2 shows the content of the therapy. The program was composed of eight sessions, once per week for 2 h. Each session included mindfulness-meditation practice, experience sharing of mindfulness practice, and lectures on mindfulness. The participants were also asked to practice mindfulness meditation for 45–60 min per day, 6 days per week, and to be mindful of daily experiences. The 8-week course of group therapy was conducted twice, with eight people participating in the first course (three men, five women; age 44.6 ± 11.9 years) and nine people participating in the second one (two men, seven women; age 36.1 ± 6.5 years). The program was identical for each course, with the exception that a 2-h silent session, led by an assistant instructor (TK), was held as an optional session a week after the eighth session in the first course. The silent session is a module during which participants practice mindfulness meditation on their own and mostly in silence. Seven of eight subjects in the first course participated in the silent session, but their post-intervention assessment responses to the scales were received before the silent session and after the eighth session, as was done in the second group. The samples were combined for analysis because there were not enough people in either group to have a sample size with sufficient statistical power.

**Table 2:** Contents of the mindfulness group therapy

Session	Theme	Purposes and contents of the session
1st	Introduction to mindfulness	Understanding the difference between “doing mode” and “being mode” through experiences of eating exercise and body scan.
2nd	Messages from body and breath	Addressing the importance and difficulty of observing experiences by being aware of body sensations.
3rd	Connecting mind with body	Being aware of the sensation of the moving body and changes in thoughts and emotions through stretching meditation. Being in touch with unpleasant feelings.
4th	Recovering own self in daily life	Sharing the daily problems with habits and learning how to switch from habitual responses to mindful coping.
5th	Letting it be	Learning non-reactive attitudes to all experiences, including the most intense experiences.
6th	Thoughts are not facts	Understanding the nature of thoughts and experiences to distance from it and be present.
7th	Caring for your own self	Accepting all aspects of yourself and learning how to choose behaviors necessary for yourself and to care yourself through compassion meditation.
8th	Making your own mindfulness program	Looking back on the 8 weeks of practices and sharing your own intentions after the program.

The group therapy was led by a main instructor (FS) and two assistant instructors. The main instructor was a clinical psychologist who had studied and practiced mindfulness meditation for more than 5 years and who had conducted 8-week mindfulness group therapy three times as a main instructor. FS had also previously participated in an MBSR program that was conducted by certified instructors. One assistant instructor (TK) was a graduate student majoring in clinical psychology who had studied and practiced mindfulness meditation for more than 5 years and had conducted an 8-week mindfulness group therapy once as a main instructor. TK had also been a participant in an MBSR program that was conducted by certified instructors. Another assistant instructor (HK) was a medical doctor and a clinical psychologist who had practiced mindfulness meditation for more than 10 years and taught mindfulness at a university.

### Assessments

At the pre- and post-intervention and 2-month FU, participants were asked to answer self-reported scales. They were refunded 500 Japanese yen if they returned answers at all three points. At the FU, they were asked to report how much formal meditation they had practiced over the 2 months following the end of the program, in addition to giving responses to the scales shown below. Additionally, only seven people participated in measurement of electroencephalogram during tasks related to mind-wandering [21] before and after the intervention. The results will not be reported in this article, because it is not a sufficient sample size to do statistical tests and it was not measured at the 2-month FU.

### Measures

#### Depression

To assess the severity of the subjects’ depression, the Japanese version of the Beck Depression Inventory-II [22] was used. High total scores indicate high levels of depressive symptoms. The validity and reliability of this scale have been confirmed [22].

#### Anxiety

To assess anxiety, the Japanese version of the State-Trait Anxiety Inventory [23] was used. We used the sub-scale measuring trait anxiety. High total scores indicate high levels of trait anxiety. The validity and reliability of this scale have been confirmed [23].

#### Mindfulness

To assess mindfulness, the Japanese version of the Five Facet Mindfulness Questionnaire (FFMQ) [24] was used; this is a standard scale to measure multidimensional mindfulness that assesses five facets: observing (e.g., “When I’m walking, I deliberately notice the sensations of my body moving”), nonreactivity (e.g., “I perceive my feelings and emotions without having to react to them”), nonjudging (e.g., “I criticize myself for having irrational or inappropriate emotions” [a reverse item]), describing (e.g., “I’m good at finding the words to describe my feelings”) and acting with awareness (e.g., “I find it difficult to stay focused on what’s happening in the present” [a reverse item]). High scores reflect high levels of each facet of mindfulness. The validity and reliability of this scale have been confirmed [24].

To assess mindfulness, the Japanese version of the Mindful Attention Awareness Scale (MAAS) [25] was also used. It is a standard scale for measuring mindfulness, in particular the aspects of attention and awareness of the present experience (e.g., “I tend to walk quickly to get where I’m going without paying attention to what I experience along the way” [a reverse item]). High total scores on MAAS indicate high levels of mindfulness. The validity and reliability of this scale have been confirmed [25].

#### Mind wandering

To assess mind wandering, the Japanese version of the Mind Wandering Questionnaire [26] was used. High total scores indicate high levels of mind wandering tendency. The validity and reliability of this scale have been confirmed [26].

#### Self-compassion

To assess self-compassion, the short form of the Japanese version of the Self-Compassion Scale (SCS) [27] was used. Although the SCS is theorized as having six-factor structures, confirmatory factor analysis of a higher two-factor model showed sufficient fit indices comparable to the six-factor model of the short form of the Japanese version of the SCS [27]. Therefore, we used a two-factor model with positive and negative factors for self-compassion. The positive factors include items directly referring to self-compassion, such as self-kindness (e.g., “When I’m going through a very hard time, I give myself the caring and tenderness I need.”), while the negative factors include items opposed to self-compassion, such as self-judgment (e.g., “I’m disapproving and judgmental about my own flaws and inadequacies.”). High total scores for the positive factors indicate high levels of self-compassion, while high total scores for the negative factors indicate low levels of self-compassion. The validity and reliability of this scale have been confirmed [27].

#### BIS/BAS

To assess BIS/BAS, the Japanese versions of the BIS/BAS scales [28] were used. High total scores for the BIS scale indicate high levels of the BIS, while high total scores for the BAS scale indicate high levels of the BAS. The validity and reliability of these scales have been confirmed [28].

### Statistical analyses

To examine changes in outcomes and process variables through mindfulness group therapy, we conducted a repeated one-way ANOVA (three levels: pre- and post-intervention and FU), and then post hoc tests were conducted, using Holm’s method. In addition, we calculated Hedge’s *g* for the mean differences between the two levels. To examine the relationships between the changes of outcome and process variables, we calculated the differences in scores (pre value – post value and pre value – FU value) and showed correlations between the differences in scores. We used Spearman’s correlation coefficient because it is less influenced by outliers. The significance tests in the correlations were not adjusted. A missing value for an item was complemented using Shimizu and Imae’s [23] procedure. The procedure was performed by dividing the sum of the scores of the answered items by the number of answered items and subsequently multiplying the obtained result by the total number of items in the subscale (e.g., if a participant answered three of five items, we first summed the scores of the answered items. We then divided that sum by the number of answered items and finally multiplied the result by the number of items for each subscale [i.e., five]) to estimate the total score for each subscale. The significance level was.05 for all analyses. Data were analyzed with the statistical software HAD 16_050 [29].

## Results

### Changes in outcome variables

Table 3 shows the results. The ANOVA showed that the main effects were significant for depression and anxiety. Post hoc tests showed significant decreases in depression and anxiety from pre- to post- intervention, and from pre-intervention to FU. Furthermore, the decrease in trait anxiety from post-intervention to FU was also significant (*adj p* = .024, *g* = .35). The average for total meditation hours for the 2 months following the end of the program was 17.9 ± 18.2 h.

**Table 3:** Means, SDs, and the results of repeated one-way ANOVAs, post hoc tests, and effect sizes

	*Mean* (*SD*)											Pre - Post (post hoc test)			Pre - FU (post hoc test)		
Variables	Pre		Post		FU		partial *η*2	*F*(2, 30)	*P* (main effect)			*g*	*t* (15)	*adj P*	*g*	*t* (15)	*adj P*
Depression	14.3	(8.3)	6.1	(4.8)	6.1	(4.5)	.489	14.3	< .001***			1.18	4.7	< .001***	1.21	3.6	.005**
Trait-anxiety	53.4	(9.5)	48.1	(10.1)	44.6	(9.2)	.459	12.7	< .001***			.53	2.5	.024*	.92	4.9	< .001***
Mindfulness FFMQ total	114.8	(20.7)	127.4	(20.0)	131.3	(20.4)	.348	8.0	.011*			−.61	−2.5	.023*	−.79	−3.1	.023*
Observing FFMQ	24.3	(5.4)	28.4	(3.7)	28.1	(4.5)	.408	10.3	.002**			−.87	−3.4	.007**	−.75	−3.5	.010*
Nonreactivity FFMQ	18.8	(4.6)	22.4	(3.7)	24.0	(4.6)	.429	11.3	.002**			−.85	−2.9	.023*	−1.11	−3.9	.005**
Nonjudging FFMQ	24.2	(7.4)	26.8	(6.6)	29.2	(5.0)	.262	5.3	.021*			−.37	−1.4	.168	−.77	−2.9	.032*
Describing FFMQ	23.3	(4.5)	24.9	(5.9)	24.9	(7.3)	.126	2.2	.149			−.31			−.27		
Acting with awareness FFMQ	24.3	(6.6)	24.8	(4.6)	25.1	(5.7)	.014	0.2	.752			−.10			−.13		
Mindfulness MAAS	56.6	(9.5)	56.0	(10.9)	59.7	(8.4)	.111	1.9	.185			.06			−.33		
Mind-wandering	17.9	(2.6)	17.4	(2.5)	16.6	(2.5)	.110	1.9	.185			.19			.48		
Self compassion total	31.7	(10.6)	35.4	(10.2)	38.4	(8.5)	.271	5.6	.014*			−.35	−1.5	.151	−.69	−3.3	.015*
Self compassion positive factor	16.6	(5.6)	18.7	(4.7)	20.3	(5.1)	.258	5.2	.014*			−.39	−1.6	.122	−.67	−3.0	.029*
Self compassion negative factor	20.9	(5.2)	19.3	(6.0)	17.9	(4.9)	.210	4.0	.036*			.28	1.3	.226	.59	2.9	.036*
BIS	22.5	(4.0)	21.2	(3.8)	20.6	(4.0)	.150	2.6	.103			.33			.46		
BAS	36.3	(4.4)	37.6	(4.3)	36.5	(4.8)	.063	1.0	.379			−.29			−.04		

***p < .001, **p < .01, *p < .05

### Changes in process variables

Table 3 shows the results. The main effects on the total scores on the FFMQ and for observing, nonreactivity, and nonjudging were significant. Post hoc tests showed that the increases in the FFMQ total were significant from pre- to post- intervention, from pre-intervention to FU, and from post-intervention to FU (*adj p* = .038, *g* = −.19). Increases in observing were significant from pre- to post- intervention and from pre-intervention to FU. Increases in nonreactivity were significant from pre- to post-intervention, from pre-intervention to FU, and from post-intervention to FU (*adj p* = .021, *g* = −.36). Increases in nonjudging were significant from pre-intervention to FU, and from post-intervention to FU (*adj p* = .037, *g* = −.40). The main effects on describing (*p* = .149) and acting with awareness (*p* = .752) were non-significant. The main effect on MAAS was also not significant (*p* = .185).

The main effect on mind wandering was not significant (*p* = .185).

For SCS, the main effects on the total score and the positive and negative factors were significant. Post hoc tests showed an increase of total SCS from pre-intervention to FU that was significant. The increase in the positive factor of SCS was significant from pre-intervention to FU. The decrease in the negative factor of SCS was significant from pre-intervention to FU.

The main effects on BIS (*p* = .103) and BAS (*p* = .379) were not significant.

### Correlations between the changes in outcome and process variables

Table 4 shows the correlations between changes from pre- to post-intervention and pre- to FU. Changes in depression and anxiety from pre- to post-intervention showed a marginally significant negative correlation with the total FFMQ score. Depression showed a marginally significant negative correlation with nonreactivity, and anxiety showed a significant negative correlation with nonreactivity. In addition, anxiety showed a significant negative correlation with describing and marginally with mindfulness, as measured by MAAS. Neither depression nor anxiety was significantly correlated with mind wandering. Both showed a significant negative correlation with total SCS score and a positive factor, while only with anxiety was there a significant positive correlation with the negative factor. In the correlations with the BIS/BAS, only anxiety showed a positive correlation with the BIS.

**Table 4:** Correlations between changes in outcome and process variables from pre to post or FU

	Pre-to post-intervention		Pre-intervention to FU	
	Depression	Trait anxiety	Depression	Trait anxiety
Mindfulness FFMQ total	−.44 +	−.48 +	−.37	−.60*
Observing FFMQ	−.40	−.16	−.42	−.37
Nonreactivity FFMQ	−.44 +	−.60*	−.25	−.35
Nonjudging FFMQ	−.28	−.40	−.55*	−.54*
Describing FFMQ	−.29	−.59*	−.12	−.44 +
Acting with awareness FFMQ	−.42	−.40	−.40	−.36
Mindfulness MAAS	−.28	−.47 +	−.32	−.31
Mind wandering	.02	.34	.14	.12
Self-compassion total	−.51*	−.76**	−.62*	−.58*
Self-compassion positive factor	−.70**	−.51*	−.56*	−.50 +
Self-compassion negative factor	.38	.83**	.54*	.68**
BIS	.26	.61*	.47 +	.48 +
BAS	.11	.03	.03	.25

Note: Process variables are the difference scores between pre- and post-intervention in the left two columns and pre-intervention to FU in the right two columns.

Correlation coefficients are Spearman’s correlation coefficients

^**^ *p* < .01, ^*^ *p* < .05, ^+^ *p* < .10

Changes in anxiety from pre-intervention to FU showed a significant negative correlation with the total scores for the FFMQ. Both depression and anxiety showed significant negative correlations with nonjudging. Furthermore, anxiety showed a marginally significant negative correlation with describing. Neither depression nor anxiety were significantly correlated with mind wandering. Both showed significant and marginally significant correlations with total SCS scores, the positive factor (negative correlation), and the negative factor (positive correlation). Both depression and anxiety showed a marginally significant positive correlation with the BIS, and neither showed a significant correlation with the BAS.

## Discussion

This study investigated three hypotheses: After doing the program, participants’ 1) depression and anxiety scores will decrease (outcome variables) and these decreases will last at least until the 2-month FU; 2) mindfulness, self-compassion, and the BAS scores will increase, and mind wandering and the BIS scores will decrease (process variables), and the changes will last at least up to the 2-month FU; and 3) changes in outcome and process variables will correlate. For hypothesis 3, we examined correlations of depression and anxiety to each process variable.

Depression and anxiety scores significantly decreased from pre- to post-intervention, with moderate to large effect sizes. Those decreases were sustained at least up to the 2-month FU. Therefore, hypothesis 1 was supported. These results are consistent with the results of meta-analysis studies, which have shown positive effects from mindfulness-meditation programs for depression and anxiety at post-intervention and FU [30, 31]. Furthermore, anxiety significantly decreased from post-intervention to FU in the current study. It may be that learning mindfulness through the application of mindfulness for daily situations and voluntary meditation practices (17.9 ± 18.2 meditation hours over the 2 months post-intervention) after the program might decrease anxiety even further. The results showed that this 8-week mindfulness program was effective for people suffering from depression and anxiety in Japan. Further study is needed to compare such a mindfulness-based program with a control intervention.

As far as the process variables were concerned, only the total scores for FFMQ, observing, and nonreactivity were increased from pre- to post-intervention with increases sustained until FU. Therefore, hypothesis 2 is only supported in regard to some aspects of mindfulness. However, nonjudging was increased from pre-intervention to FU and post to FU. Thus, it is possible that the practice of mindfulness through daily application or formal practice after the program could contribute to delayed improvement. This is consistent with further improvements in trait anxiety from post-intervention to FU. However, describing, acting with awareness, and mindfulness as measured by MAAS, which included overlapping items with acting with awareness, unexpectedly did not increase. Acting with awareness did not significantly increase in similar studies of 8-week mindfulness programs in Japan [5, 32]. It is possible that the Japanese response style to acting with awareness and MAAS, which inquire as to the degree one’s own awareness of one’s current state, is distinct from that of other cultural populations. However, the results for describing are inconsistent with those of another study in Japan, where describing significantly increased before and after a mindfulness-based program [5]. The results may depend on the variant characteristics of the participants, instructors, and also on the content of the program. The describing scores (pre-intervention, 23.3 ± 4.5; post-intervention, 24.9 ± 5.9) in our sample were higher than those (pre-intervention, 19.3 ± 6.2; post-intervention, 21.2 ± 5.6) obtained by Yanagisawa et al. [5]. Further, the participants were more varied in Yanagisawa et al. [5], whose study included people who were, for example, diagnosed with schizophrenia, fibromyalgia, or hypochondria. Therefore, it is possible that our participants evinced relatively fewer problems with regard to the skill of describing their own experiences did than the previous study. With regard to the content of the program, Yanagisawa et al. [5] conducted some role plays and experience sharing about how to deal with thoughts and emotion in addition to following the MBCT manual [3]. Also, the average number of participants was 4.7 per course, which amounted to relatively fewer people and provided participants with more opportunities to describe and to report their experience in a session. Those differences may have affected the differences in the changes recorded in the describing scores between our study and Yanagisawa et al. [5].

Self-compassion increased from pre-intervention to FU but not from pre- to post-intervention. This is inconsistent with Bergen-Cico and Cheon [33], who showed that self-compassion significantly increased through mindfulness-based intervention for full-time and part-time students in the USA. However, Ito, Sasara, Kuriyama, Kikai, Hiranaka, Tamae, and Sakamoto [32] did not find a significant increase in self-compassion through mindfulness-based intervention for professional palliative caregivers in Japan. Although the samples for those studies differ from those of the current study, it may be that Japanese participants have difficulty learning self-compassion through a mindfulness-based intervention, at least as assessed soon after the program. Despite the delay in the effect, the increase from pre-intervention to FU is in line with the finding that demonstrated an increase in self-compassion from the pre-intervention stage to 1-month after the MBCT for recurrent depression [15].

Furthermore, mind wandering was not significantly changed. It may be that mind wandering is distinct from negative repetitive thinking in its perseveration. Negative repetitive thinking like rumination and worry have shown mediator effects in 8-week mindfulness programs [6, 7]. It may be more important to decrease the perseverative aspects of thinking in 8-week mindfulness programs. To determine this, measurement of mind wandering and perseverative thinking must be conducted concurrently, and the mediating effects must be compared.

For the BIS/BAS, there were no significant changes. Changing such an aspect of temperament as the BIS/BAS may be difficult in an 8-week program. Furthermore, BAS activity is said to cause beginning (or increasing) movement toward goals. This is seemingly inconsistent with the “being mode,” which does not drive an individual to capture something, as taught in mindfulness programs. The BAS scale may not discriminate positive feelings directed by mindfulness from hedonic feelings related to the “doing mode.”

Improved nonreactivity was correlated with decreased depression and anxiety from pre- to post-intervention, as predicted in hypothesis 3. Furthermore, improved nonjudgment was also correlated with decreased depression and anxiety from pre-intervention to FU. Nonreactivity and nonjudging belong with the acceptance component in Monitor and Acceptance Theory [34], a recently proposed model to describe mechanisms of mindfulness for cognition, affect, stress, and health. Our results showed that the acceptance component is important for effects on depression and anxiety as a common mechanistic variable. In FFMQ, nonreactivity includes positive items, while nonjudging has only reversed items. It is possible that nonreactivity was actively trained within the 8-week program, while arising judgment reduced in delayed timing. Thus, nonreactivity is effective for symptoms within the program period, while nonjudging might be effective in the period that includes FU. Describing was specifically correlated with trait anxiety. It might be an important factor for people who have anxiety to notice their experiences and express them in particular.

Self-compassion had a relatively large correlation with depression and anxiety as a whole. This is consistent with studies showing a mediating effect for self-compassion in the effects of MBCT on recurrent depression [15]. It is interesting that the only positive factor for SCS is correlated with depression from pre- to post-intervention, even though the negative factor for SCS includes some items that are similar to depressive symptoms, for example, a sense of isolation. These results imply that self-compassion is closely related to depression and anxiety and that it has a high potential to improve those symptoms.

Finally, decreased BIS was correlated with improved anxiety and depression (only from pre-intervention to FU). The BIS may cause avoidance, which is a primary target of mindfulness-based intervention. BIS items include covert avoidance, which might be attenuated by mindfulness intervention. In sum, hypothesis 3 is supported regarding some aspects of mindfulness, self-compassion, and the BIS.

## Limitations

The lack of a control condition reduces the ability to ensure that the mindfulness program caused the changes in the outcome and process variables. It is possible that placebo effects and Hawthorne effects were confounded and may be relatively large, given that mindfulness is becoming popular in Japan. We could not detect small or moderate effects because of the sample size. The instructors were not certified teachers of either MBSR or MBCT, which could lead to some inconsistency with previous studies. All the measurements included self-reported scales, and these may be biased, as with any case of measuring mindfulness by self-report [35]. Future studies are needed to resolve these problems and confirm the effects of 8-week mindfulness programs while examining action mechanisms. Furthermore, the participants of this study presented mild to moderate depression or anxiety at pre-intervention, so we cannot generalize our conclusions to people with severe symptoms. In addition, the symptom levels and the variety of diagnoses were wide-ranging. Future studies with more rigid inclusion assessments are needed to clarify the population for which mindfulness group therapy is effective.

## Conclusions

We found that depression and anxiety scores were significantly decreased after participation in an 8-week mindfulness group therapy for depressive and anxious people in Japan. The decrease in depression scores was sustained and anxiety scores decreased further from post-intervention to 2-month FU. Those results show the efficacy of this 8-week mindfulness group therapy for depression and anxiety in Japan and indicate the feasibility of further randomized controlled trials. Among process variables, some facets of mindfulness significantly increased from pre- to post-intervention, and those increases were sustained until FU. Only self-compassion significantly increased from pre-intervention to FU, while mind wandering and BIS/BAS did not change. Improvements in certain facets of mindfulness and self-compassion were significantly correlated with decreases in depression and anxiety. Therefore, mindfulness and self-compassion may be important mediators for the effects of an 8-week mindfulness group therapy program, which is consistent findings of previous studies.

## Abbreviations

ANOVA: Analysis of variance
BAS: Behavioral activation system
BIS: Behavioral inhibition system
DSM: Diagnostic and statistical manual of mental disorders
FFMQ: Five Facet Mindfulness Questionnaire
FU: Follow-up
MAAS: Mindful Attention Awareness Scale
MBCT: Mindfulness-based cognitive therapy
MBSR: Mindfulness-based stress reduction
M.I.N.I.: Mini-International Neuropsychiatric Interview
SCS: Self-Compassion Scale
